# Case report: Diagnostic and therapeutic challenges of fungal endocarditis by *Trichosporon asahii* in a child with congenital heart defects

**DOI:** 10.3389/fped.2023.1200215

**Published:** 2023-10-06

**Authors:** Amanda Baptistella, Ana Júlia A. Rossato, Beatriz C. de Gusmão, Carolina M. Cunha, Luiza F. Trafane, Paulo C. M. Colbachini

**Affiliations:** ^1^ Pontifical Catholic University of Campinas School of Medicine, Campinas, Brazil; ^2^ Pediatric Residence Program, Pontifical Catholic University of Campinas Hospital, Campinas, Brazil; ^3^ Pediatric Intensive Care Residence Program, Pontifical Catholic University of Campinas Hospital, Campinas, Brazil

**Keywords:** fallot’s tetralogy, prosthetic valve, infective endocarditis, *Trichosporon asahii*, case report

## Abstract

**Background:**

patients with congenital cardiopathies are the main group at risk for infective endocarditis (IE) in the pediatric population. Fungal etiology is responsible for 2%–4% of all IEs, and the *Trichosporon* genus is an increasingly prevalent cause of infections in human beings.

**Case presentation:**

We describe a 9-year-old male with multiple surgical procedures to correct congenital cardiopathy defects, including insertion of RV-PA conduit, who was admitted due to suspicion of pneumonia and needed a surgical approach after being diagnosed with a mycotic pseudoaneurysm in the right ventricle’s outflow tract, with dilation of the RV-PA conduit. The conduit was removed and antifungal treatment was started with Voriconazole after the agent was identified (*T. asahii*), with satisfactory therapeutic response. Approximately 4 years later, the patient was readmitted, presenting with intermittent fever, associated with nocturnal diaphoresis, dry cough, anxiety and chest pain. Vegetations consistent with *T. asahii* were evidenced in the RV-PA conduit, and a surgical approach was once again necessary.

**Discussion:**

diagnostic methods and treatment of *T. asahii* endocarditis aren't yet standardized, and recurrent surgical approaches are needed due to the inefficacy of antifungal treatment.

## 1. Introduction

*Trichosporon* are yeast-like basidiomycetes, and *T. asahii* is known as the most pathogenic amongst this genus. Infections by *T. asahii* are an emerging, although rare, clinical entity, especially considering immunocompetent patients without hematologic malignancies (1). The incidence of systemic *Trichosporon* infections (trichosporonosis) in the context of heart disease is usually limited to transplant patients who are under immunosuppressive therapy (2). The origin of these infections, especially post-operatively, is still uncertain (3).

Regardless of the improvements in pharmacological and surgical therapies, fungal infective endocarditis (IE) is still associated with a poor prognosis and remains a diagnostic and therapeutic challenge (4). Risk factors for fungal endocarditis in children include surgical procedures which require implantation of prosthetic devices, prolonged intravenous catheterization, multiple broad-spectrum antibiotic use, parenteral nutrition and immunosuppression.

Due to the relevance of the subject, this paper reports the case of a school-aged child with congenital heart disease admitted to our service with recurrence of trichosporonosis after 4 years, suggesting an inadequate response to the initial therapeutic approach.

## 2. Case report

**Figure 1:**
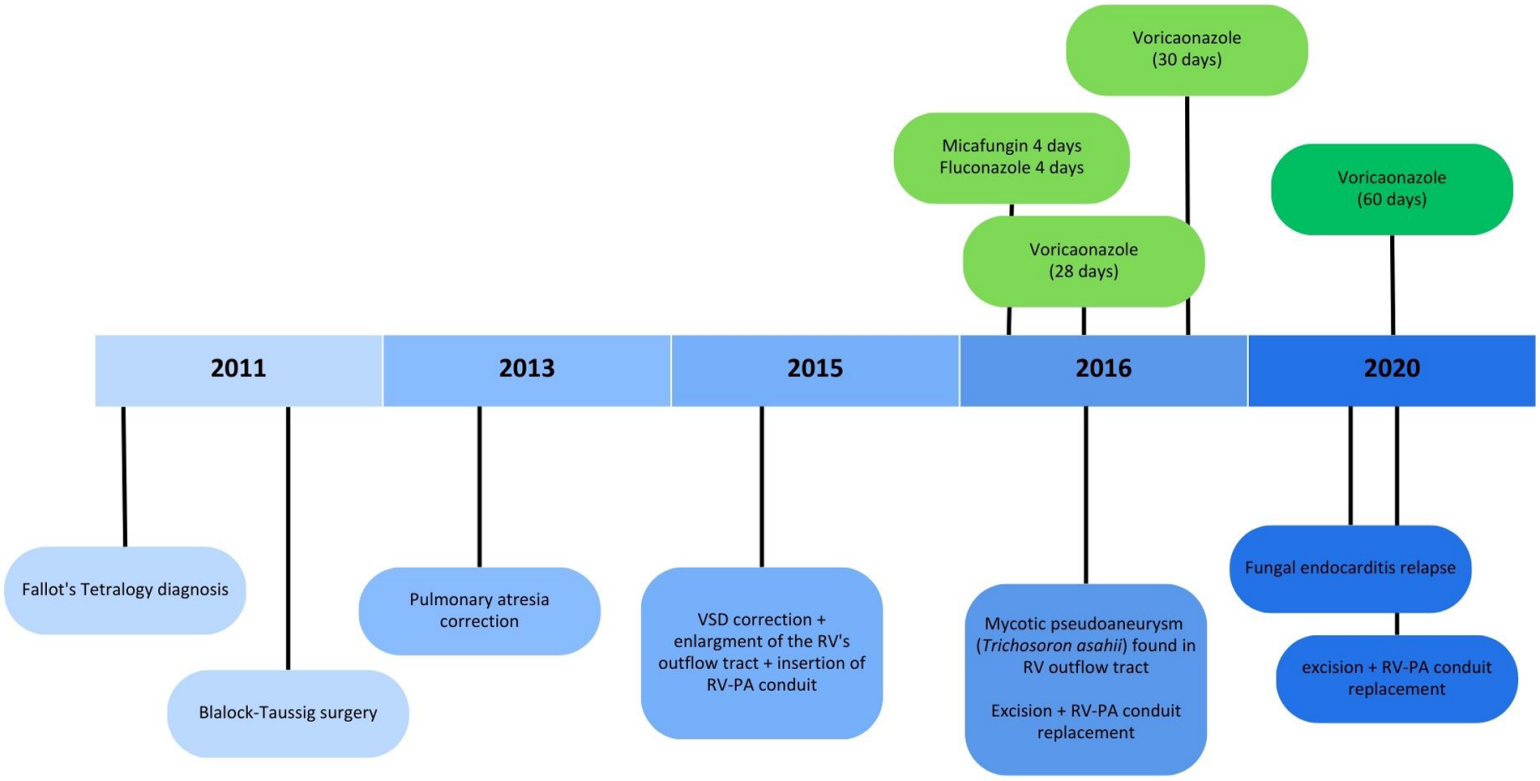
Timeline presenting most relevant episodes of the case report (VSD, ventricular septal defect; RV, right ventricle; RV-PA, right ventricle-pulmonary artery).

**Figure 2:**
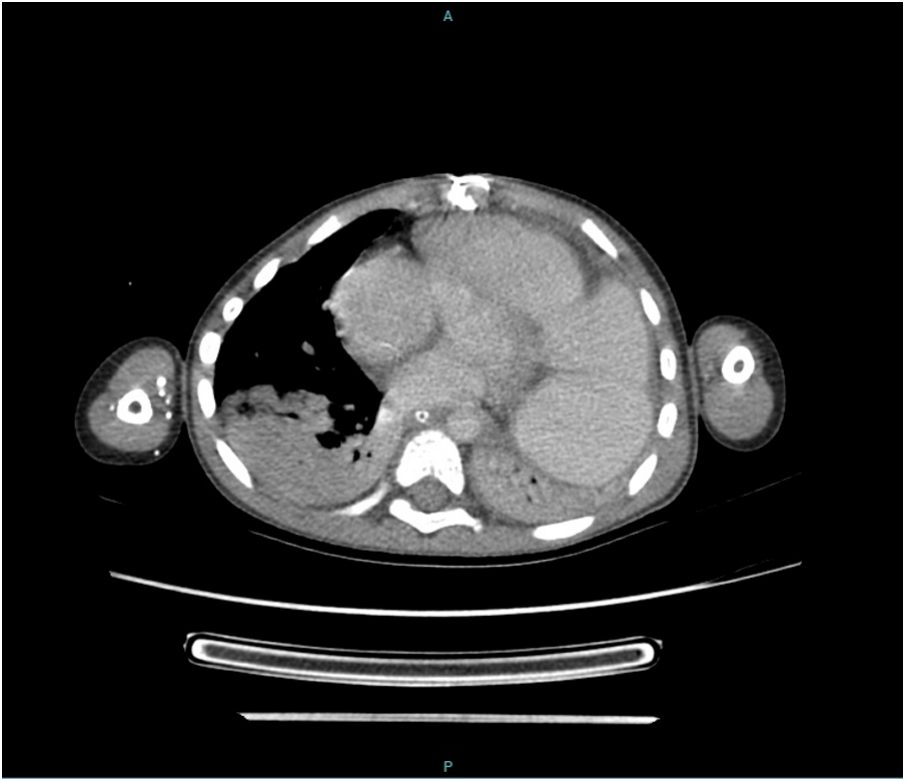
CT scan showing dilation of the RV-PA conduit (and also right and left lower lobes atelectasis).

We report the case of a 9-year-old male patient with a history of recurrent pulmonary infections and multiple operations to correct congenital heart defects (Figure 1). The patient was diagnosed with Fallot’s Tetralogy at birth, and had a Blalock-Taussig surgery 3 days after birth, correction of pulmonary atresia at age 2 and correction of Ventricular Septal Defect (VSD), associated with enlargment of the right ventricle’s outflow tract and insertion of an RV-PA conduit at age 4. Ten months after the last surgical intervention, the patient was admitted to the Emergency Department presenting with dyspnea and respiratory insufficiency, and was admitted due to suspicion of pneumonia. CT scan didn't show pulmonary involvement, but revealed dilation of the RV-PA conduit (Figure 2), and the patient was taken to surgery. Vegetations consistent with mycotic pseudoaneurysm were found in the outflow tract of the right ventricle, and the conduit was excised. Empirical antifungal treatment was started with micafungin for 4 days, with no clinical improvement, followed by fluconazole for 4 days. After this 8-day period, cultures from a sample of pleural fluid and of fragment of the conduit removed in surgery were positive for *Trichosporon asahii*. Both samples were cultured on Sabouraud dextrose agar and blood agar medium, but the time of positivity was not described, and susceptibility test was not performed for either samples. Species was identified using VITEK ® identification system with specific cards for fungi and yeasts identification, but unfortunately no images of the colonies were taken. After this, Voriconazole was started and kept for 28 days and the patient responded well.

**Figure 3:**
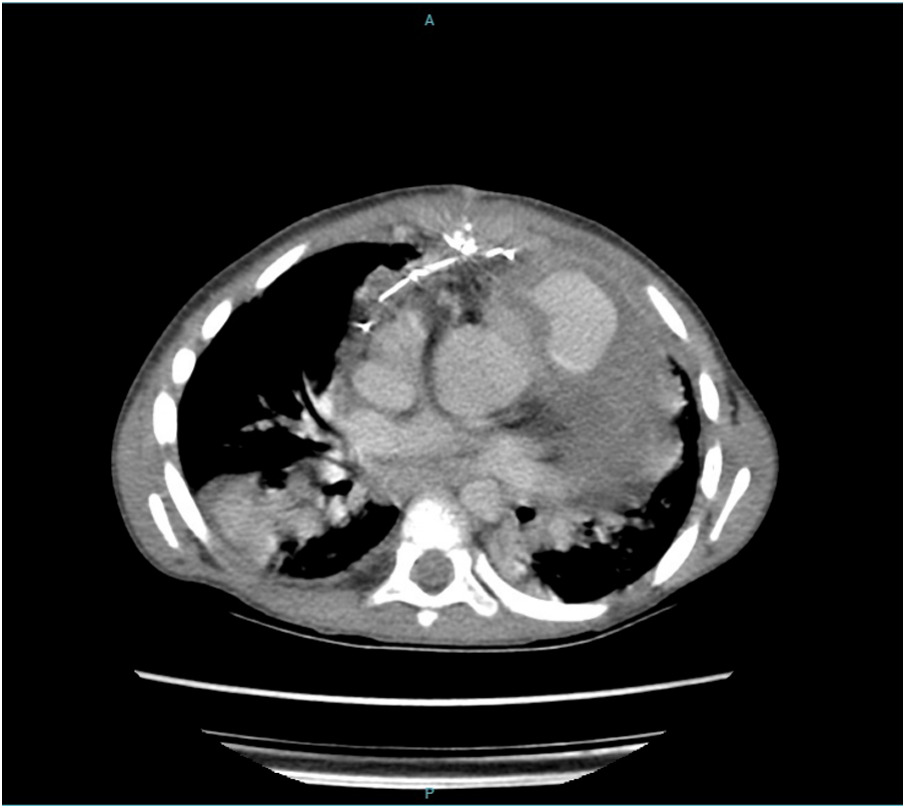
CT scan showing images compatible with pulmonary necrosis in the upper segment of the right lower pulmonary lobe.

One month later, he presented with clinical deterioration, and a new CT scan showed images compatible with pulmonary necrosis in the upper segment of the right lower pulmonary lobe (Figure 3). The patient went under thoracotomy with segmentectomy of the middle lobe of the right lung. During the procedure, a pulmonary abscess was identified in the medium lobe. Biopsy showed the presence of septate hyphae with dichotomous branching, suggesting aspergillosis. After 17 days of admission, the patient was released with his usual drug regimen (Digoxin and Furosemide) along with Voriconazole for 30 days.

**Figure 4:**
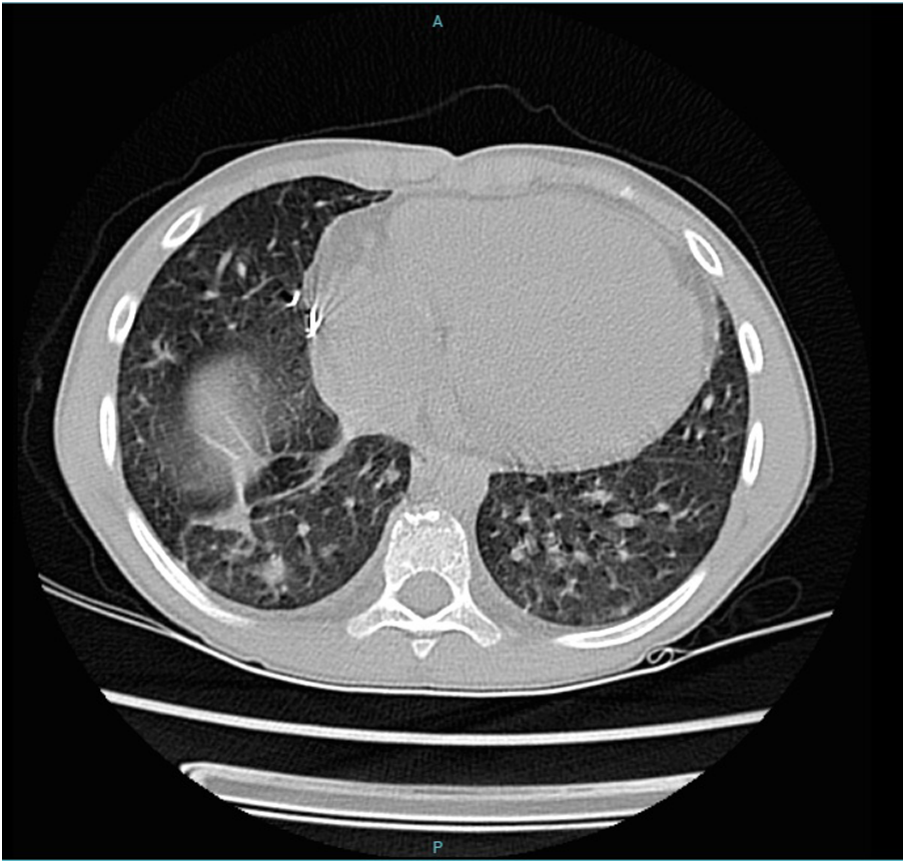
CT scan showing vegetation in the RV-PA conduit.

Four years later, at the age of 9, he was readmitted to our service presenting intermittent fever associated with nocturnal diaphoresis, dry cough, anxiety, anorexia and chest pain with a respiratory distress pattern. He was admitted for investigation, and a CT scan once again evidenced vegetation in the RV-PA conduit (Figure 4). Once more, cultures were positive for *T. asahii*, this time two blood samples cultured on Sabouraud and blood agar medium, which tested positive after 7 and 8 days, respectively. Again, VITEK® was used for identification and susceptibility testing was not performed. Antifungal treatment was once again started with Voriconazole and the patient was sent into surgery to exchange the RV-PA conduit. Voriconazole therapy was kept for two months. The drug was suspended when blood cultures were negative for *T. asahii*. The patient progressed well, with no other episodes of fever or other complaints.

## 3. Discussion and conclusion

Fungal infections caused by the *Trichosporon* genus are knowingly on the rise as a clinical entity (1). The gravity of the infections caused by *Trichosporon spp*, the late diagnosis and the lack of standardized antifungal treatment contribute to the high mortality rates in this scenario (5). Among this genus, *T. asahii, T. asteroides* and *T. mucoides* present with increased pathogenic potential. *T. asahii* is highlighted as the major agent in invasive infections and is strongly associated with poor prognosis, possibly due to its reduced sensibility to azoles (1). Invasive infections are related to neutropenic patients, immunosuppressive therapy in transplant patients or chronic corticosteroid use, newborns, patients with autoimmune disorders and the period following very debilitating illnesses, such as trauma, burns and surgeries (6, 7). The mortality rate of patients with *T. asahii* infective endocarditis can reach 82%, representing a very poor prognosis (2).

Despite preventive strategies, the origin of Trichosporonosis is still uncertain. It is known that *T. asahii* is a component of the gastrointestinal and skin microbiomes in human beings (8, 9) and slight alterations in the microenvironment can activate its pathogenic potential, leading to the subversion of immune tolerance and consequent tissue damage and fungemia, enabling systemic infection (10). Furthermore, *T. asahii* can form biofilm in prosthetic valves or implants, which also contributes to the progression of invasive postoperative infections and increases the resistance rates to antifungal agents and the host’s immune response (11).

There are very few reported cases of patients with complicated fungal endocarditis caused by *Trichosporon asahii* in literature. The first case report was published in 2009. A 58-year-old male admitted due to high fever and weight loss was diagnosed with endocarditis associated with mitral and aortic vegetations. Nine days after admission, cultures were positive for *T. asahii.* The treatment chosen in this case was fluconazole 400 mg/day and double valve replacement 34 days after admission. After surgery, the treatment with fluconazole was sustained and associated with ampicillin and gentamicin. The fever stopped and cultures were negative for *T. asahii* after this period. The patient also tested negative for Beta-D glucan. 7 weeks after the surgery, the dosage of fluconazole was reduced to 200 mg/day, without projection of suspension. He was released 85 days after admission. Until the moment this article was written, there were no other cases in the literature reporting fungal endocarditis by *T. asahii* associated with fungemia and treated with surgical approach (2).

Infective endocarditis represents more than a therapeutic challenge—it is still a diagnostic challenge. Duke’s criteria lack sensibility in fungal endocarditis. A high suspicion index is needed to corroborate with the diagnosis of fungal etiology, enabling the early start of therapeutic measures, which knowingly improve the prognosis (12). Echocardiography has a pivotal role in the diagnosis and management of Infective Endocarditis, and is the technique of choice for diagnosis and management. In cases of initial investigation, the results can be inconclusive or normal in up to 30% of the cases. Transesophageal tridimensional echocardiography improved the evaluation of the volume of cardiac chambers, particularly to identify paraprosthetic reflux. Other imaging methods, such as MRI, multislice computer tomography, and PET/CT, also could can aid in the diagnosis (13).

The difficulty of diagnosis also reflects in negative blood cultures, which occur in about 2%–20% of endocarditis cases. The usual causes for false negatives are concomitant or previous use of antibiotics and the presence of microorganisms with slow growth or with difficulty detect in routine cultures, among them fungi stand out. Definitive diagnosis goes far beyond blood culture and depends on the culture or histology of the vegetation, followed by the isolation of microorganisms and subsequent identification as *Trichosporon asahii* (1).

Treatment includes the immediate institution of antifungal therapy, prosthesis replacement and long-term eradication therapy. However, there are no studies that determine the correct duration of antifungal treatment in these cases (12). When trichosporonosis is defined, recurrence is common even with continued antifungal treatment for up to 2 years (14). Fungi of *Trichosporon spp*. genus, in addition to forming biofilms that help with resistance to antifungal drugs, also produce proteases and lipases that increase their virulence, such as beta-N Acetyl Hexosaminidase and glucuronoxylomannan expression in their cell walls, a polysaccharide capable of attenuating the phagocytic capacity of *in vivo* neutrophils and monocytes (11). Thus, the optimal pharmacological treatment options for trichosporonosis are not yet well established, but it is already known that there is reduced sensitivity to some azole drugs. These fungi also present with greater resistance to flucytosine and variable susceptibility to amphotericin B, with relative resistance to this agent being observed. However, most studies have shown that triazoles have the lowest values of minimum inhibitory concentration (MIC) for these fungi, especially voriconazole, in addition to these having greater activity *in vitro* compared to amphotericin B (1, 5, 15).

Susceptibility tests, despite its potential to predict clinical success, this may be diminished in the presence of highly complex infections, and resistance may not necessarily correlate with treatment failure (16). Furthermore, the methodologies employed to delineate susceptibility profiles for *Trichosporon spp*. have not been extensively accessed (17), and there are no established breakpoints to define antifungal susceptibility for this species (18). Nevertheless, susceptibility tests are important as diagnostic tools, since they allows clinicians to tailor antimicrobial therapy thereby maximizing treatment efficacy and minimizing the development of resistance, and for most invasive fungal infections are routinely recommended (16). In the case we have reported, susceptibility tests were not performed due to the lack of standardization for *Trichosporon* susceptibility tests in the Brazilian Committee on Antimicrobial Susceptibility Testing (BrCAST—EUCAST). Thus, treatment with Voriconazole was empirically chosen based on its documented superior efficacy in the literature.

Voriconazole is a large-spectrum triazole agent derived from fluconazole. Studies have shown that a large part of its fungicidal action against *Trichosporon* is due to two main factors: its high affinity for 14-alpha-demethylase fungi and the inhibition of 24-methylene-dihydrolanosterol demethylation of some yeasts and filamentous fungus. Therefore, many authors use triazoles, alone or in combination, as drugs of choice for the treatment of *Trichosporon* fungemia (15, 19). Voriconazole has been shown to have good activity against *Trichosporon* species (12, 20–21). Tsai et al., testing the species susceptibility to Amphotericin B, Fluconzazole and Voriconazol, found in a sample of (22) *T. asahii* isolates, that MIC_50_ and MIC_90_ for Voriconazole were 0.031 and 0.063 µg/ml respectively, against 0.25 and 1 µg/ml, respectively for Amphotericin and 2 and 4 µg/ml for Fluconazole, although “no unanimous MIC of amphotericin B and voriconazole was obtained among different methods” (both EUCAST and CLSI) (22). In another series, susceptibility results demonstrated by Rodriguez-Tudela et al. showed that most of their *T. asahii* isolates had Amphotericin B geometric mean (GM) MICs >4 µg/ml versus GM MIC ≤0.14 µg/ml for Voriconazole (regardless the species of *Trichosporon* analyzed) (23). Furthermore, Ruan et al. described Voriconazole as the most potent drug tested against *Trichosporon* (MIC ≤0.5 µg/ml) outperforming eight other antifungal agents, with good *in vitro* activity even against isolates resistant to Fluconazole (24). Similary, Kuo et al., although not utilizing CLSI or EUCAST methodologies for their susceptibility tests, also identified Voriconazole as “the most potent agent *in vitro*” for *Trichosporon* species with a GM MIC of 0.111 µg/ml (25). Lastly, Chagas-Neto et al. described that most of the *T. asahii* isolates in their series had high MICs for Amphotericin B (≥2 µg/ml), Caspofungin (≥2 µg/ml) and 5-Flucytosine, but all were susceptible to Voriconazole with MICs ≤0.06 µg/ml (26).

There are few cases of fungal endocarditis relapse in the literature. A case of an adolescent patient was reported with recurrence by *Trichosporon capitatum* in 1981. The patient was born with a VSD, and at the age of 14 he went through surgery to correct the VSD and to correct the pulmonary atresia and the collateral circulation. After 9 months, he started to have intermittent fever and weight loss with worsening of the symptoms. He was diagnosed with a right pulmonary artery aneurysm: during the surgery, the transplanted pulmonary valve was destroyed and the tissue was inflamed. Due to this, a new aortic graft was established and a right pneumonectomy was done. *Candida guilliermondii* was isolated from the graft. After 2 years using co-trimoxazole at home, he died of what seemed to be pneumonia. At necropsy, they found vegetations on the cusps of the graft valve. The histological examination revealed fungal hyphae and the *Trichosporon capitatum* was isolated, a fluorocytosine-resistant microorganism (27). No other similar cases that could match the case initially reported were found.

The limited data that available in the literature do not allow the standardization of diagnostic methods and treatment of *T. asahii* endocarditis, in addition to not allowing a broad study due to the lack of case reports of patients with reincident after cardiac surgery. The antifungals currently available are triazoles and amphotericin B, with evidence suggesting the superiority of triazoles; but surgery is necessary due to the ineffectiveness of these drugs alone, which contributes to the bad prognosis of this trichosporonosis, with high mortality rates. In addition, some authors suggest that the prescription of an azole derivative should be considered for life after the surgical procedure, due to the high rate of recurrence (28). In this particular case, prolonged antifungal therapy did not prevent recurrence, and surgical management was necessary to grant a positive outcome.

## Data Availability

The datasets presented in this article are not readily available because information has been summarized from the patient's medical record. Those records are protected by law and are not available to individuals from outside our institution. Requests to access the data should be directed to pc.pedpucc@gmail.com. Requests will analyzed in a case-by-case basis and provided if possible.
